# P-389. Switching to Doravirine/Islatravir (100 mg/0.25 mg) Once Daily Maintains Viral Suppression Through Week 48 in the Presence of Archived NNRTI Resistance-Associated Mutations or M184I/V in Proviral DNA

**DOI:** 10.1093/ofid/ofaf695.606

**Published:** 2026-01-11

**Authors:** Tracy L Diamond, Anjana Grandhi, Monica Fuszard, Yayun Xu, Uche Nwoke, Ying Zhang, Stephanie O Klopfer, Karen Eves, Jaime Benner, Mengchun Li, Wayne Greaves, Rima Lahoulou, Jason Y Kim, Michelle C Fox, Ernest Asante-Appiah

**Affiliations:** Merck & Co., Inc., Rahway, NJ; Merck & Co., Inc., Rahway, NJ; Merck & Co., Inc, Rahway, New Jersey; Merck, Edison, New Jersey; Merck & Co., Inc., Rahway, NJ; Merck & Co., Inc., Rahway, NJ; Merck & Co., Inc., Rahway, NJ; Merck & Co., Inc., Rahway, NJ; Merck & Co., Inc., Rahway, NJ; Merck & Co., Inc., Rahway, NJ; Merck Research Labs, Edison, NJ; MSD, Puteaux, Haute-Normandie, France; Merck & Co., Inc., Rahway, NJ; Merck & Co., Inc., Rahway, NJ; Merck & Co,. Inc, Kenilworth, NewJersey

## Abstract

**Background:**

Doravirine/islatravir [DOR/ISL (100/0.25mg)] is an investigational once-daily single tablet regimen of DOR, an approved non-nucleotide reverse transcriptase inhibitor (NNRTI), and ISL, a novel nucleoside reverse transcriptase translocation inhibitor (NRTTI), being evaluated in 3 Phase 3 studies (P051: NCT05631093; P052: NCT05630755; P054: NCT05766501) in adults living with HIV-1. Participants with HIV-1 RNA < 50 copies/mL and no known treatment failure or documented DOR resistance were randomized (2:1) to switch to DOR/ISL (100/0.25mg) once-daily or to continue baseline antiretroviral therapy (bART) in P051 or BIC/FTC/TAF in P052. In P054, participants who previously received DOR/ISL (100/0.75mg) were switched to DOR/ISL (100/0.25mg) in a single-arm study. This analysis examined the prevalence and impact of preexisting resistance-associated mutations (RAMs) at baseline in proviral DNA on virologic outcomes among participants who received DOR/ISL through Week 48 in P051, P052, and P054.

**Table 1:**
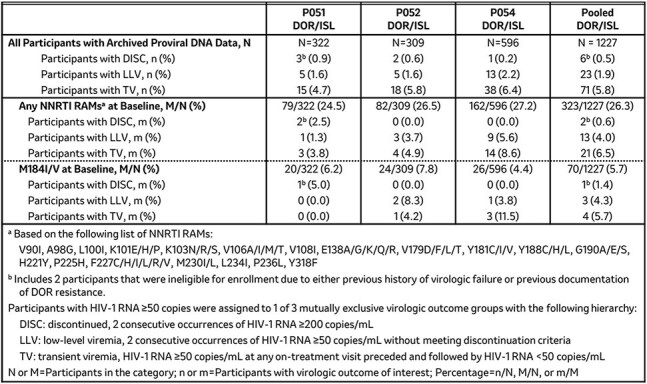
Virologic Outcomes in Participants with Preexisting NNRTI RAMs or M184I/V

**Methods:**

Preexisting RAMs present in proviral DNA were identified retrospectively using the GenoSure Archive assay (Monogram Biosciences). The impact of NNRTI RAMs and M184I/V was evaluated in participants with HIV-1 RNA ≥ 50 copies/mL at any time through Week 48 using the following virologic outcome categories: discontinued with confirmed HIV-1 RNA ≥ 200 copies/mL (DISC); low-level viremia (LLV, confirmed HIV-1 RNA ≥50 copies/mL without meeting discontinuation criteria); transient viremia (TV, HIV-1 RNA ≥ 50 copies/mL at any on-treatment visit preceded and followed by HIV-1 RNA < 50 copies/mL).

**Results:**

Of the 1227 participants with baseline resistance data who received DOR/ISL, 6 (0.5%), 23 (1.9%), and 71 (5.8%) met the criteria for DISC, LLV, or TV, respectively (Table 1). Among participants who received DOR/ISL, 26.3% had preexisting NNRTI RAMs and 5.7% had preexisting M184I/V. The percentage of participants in each virologic outcome category was similar between all participants and those with NNRTI RAMs or M184I/V.

**Conclusion:**

Preexisting NNRTI RAMs or M184I/V in proviral DNA did not impact virologic suppression through Week 48 in participants switching to DOR/ISL (100/0.25mg) in Phase 3 clinical studies.

**Disclosures:**

Tracy L. Diamond, PhD, Merck & Co., Inc.: Full-time employee|Merck & Co., Inc.: Stocks/Bonds (Public Company) Anjana Grandhi, PhD, Merck & Co., Inc.: Employment|Merck & Co., Inc.: Stocks/Bonds (Public Company) Monica Fuszard, MS, Merck & Co.: Employment Uche Nwoke, MS, Merck & Co., Inc.: Stocks/Bonds (Public Company) Ying Zhang, PhD, Merck&Co.: employee Stephanie O. Klopfer, PhD, Merck & Co., Inc: Employment|Merck & Co., Inc: Stocks/Bonds (Public Company) Karen Eves, BS, Merck & Co., Inc.: employee|Merck & Co., Inc.: Stocks/Bonds (Public Company) Mengchun Li, MD, Merck & Co., Inc.: Stocks/Bonds (Public Company) Wayne Greaves, MD, Merck & Co., Inc.: Stocks/Bonds (Public Company) Rima Lahoulou, n/a, MSD: Employment|MSD: Stocks/Bonds (Private Company) Jason Y. Kim, MD, MSCE, Merck & Co.: Employment|Merck & Co.: Stocks/Bonds (Public Company) Michelle C. Fox, MD, Merck & Co., Inc.: Employment|Merck & Co., Inc.: Stocks/Bonds (Public Company) Ernest Asante-Appiah, PhD, Merck & Co. Inc: Employee|Merck & Co. Inc: Stocks/Bonds (Private Company)

